# Low-keV virtual monoenergetic images with rapid kilovoltage-switching DECT for differentiating complicated from uncomplicated appendicitis in adults

**DOI:** 10.1007/s00261-025-05124-2

**Published:** 2025-07-17

**Authors:** Dhanawin Wongsaengchan, Jitti Chatpuwaphat, Shanigarn Thiravit, Sasima Tongsai, Napakadol Noppakunsomboon, Rathachai Kaewlai

**Affiliations:** Faculty of Medicine Siriraj Hospital, Mahidol University, Bangkok, Thailand

**Keywords:** Adult, Appendicitis, Retrospective studies, Tomography, X-Ray computed, Dual energy

## Abstract

**Objectives:**

Low-keV virtual monoenergetic images on dual-energy CT (DECT) enhance iodine attenuation in inflamed appendiceal walls, but the role in differentiating complicated from uncomplicated appendicitis remains unclear. This is particularly relevant given the shift toward nonoperative management of uncomplicated appendicitis.

**Methods:**

Consecutive adult patients with pathologically confirmed acute appendicitis who underwent preoperative rapid-kVP-switching DECT and appendectomy within 24 h were retrospectively included. Two radiologists reviewed DECT images, including a finding of appendiceal wall enhancement defects, using three series: 50-keV monoenergetic, 120-kVp-equivalent, and combined series, with discrepancies resolved by a third radiologist. Diagnostic performance of three series for differentiating complicated from uncomplicated appendicitis was assessed. Detection rates of appendiceal wall enhancement defects and radiologist confidence among three series were compared.

**Results:**

Among 155 patients (50 men, mean age 47.4±19.0 years), 59 had complicated appendicitis. The combined 50-keV/120-kVp-equivalent series provided balanced sensitivity (83.1%) and specificity (86.5%), with an accuracy of 85.2% in differentiating uncomplicated and complicated appendicitis. Although 50-keV images revealed the most wall enhancement defects (48/122; 39.3%), radiologists’ confidence was significantly higher using the combined series (91.8% vs. 72.1%, *p* < 0.001).

**Conclusions:**

Low-keV virtual monoenergetic DECT, when combined with 120-kVP-equivalent images, improved the detection of appendiceal wall enhancement defects and increased radiologist confidence in differentiating complicated from uncomplicated appendicitis in adult patients with acute appendicitis.

**Supplementary Information:**

The online version contains supplementary material available at 10.1007/s00261-025-05124-2.

## Introduction

Acute appendicitis remains the most prevalent surgical cause of acute abdominal pain in adults worldwide, with computed tomography (CT) frequently utilized for diagnosis and assessment of complications. Traditionally, appendectomy has been the standard treatment; however, a paradigm shift toward nonoperative management for uncomplicated cases has increased the need for accurate differentiation between uncomplicated and complicated appendicitis [1]. CT plays a critical role in this distinction, as the presence of complications often dictates the necessity of surgical intervention.

Dual-energy CT (DECT), particularly low-keV virtual monoenergetic imaging (VMI), offers enhanced iodine attenuation, allowing improved visualization of vascularity and tissue perfusion [2], which has been shown in the context of gangrenous cholecystitis [3, 4], bowel ischemia [5], and perforation [6]. Prior studies have reported limited sensitivity, specificity and accuracy of single-energy CT in distinguishing complicated from uncomplicated appendicitis [7, 8, 9]. However, data remain limited regarding the role of DECT for this purpose [10, 11], including its diagnostic performance, radiologist confidence, and its value in specific situations where no obvious complications, such as extraluminal air or fluid collection were identified in the light of appendicitis. Using a single-source, rapid-kVp-switching DECT system, this study aimed to quantify the diagnostic performance of DECT utilizing 50-keV images in differentiating complicated from uncomplicated appendicitis. It also evaluated clinical and DECT features independently predictive of complicated appendicitis and identified DECT findings independently predictive of complications among the subset of appendicitis patients without extraluminal air or fluid collection.

## Materials and methods

### Study design and subjects

**Fig. 1:**
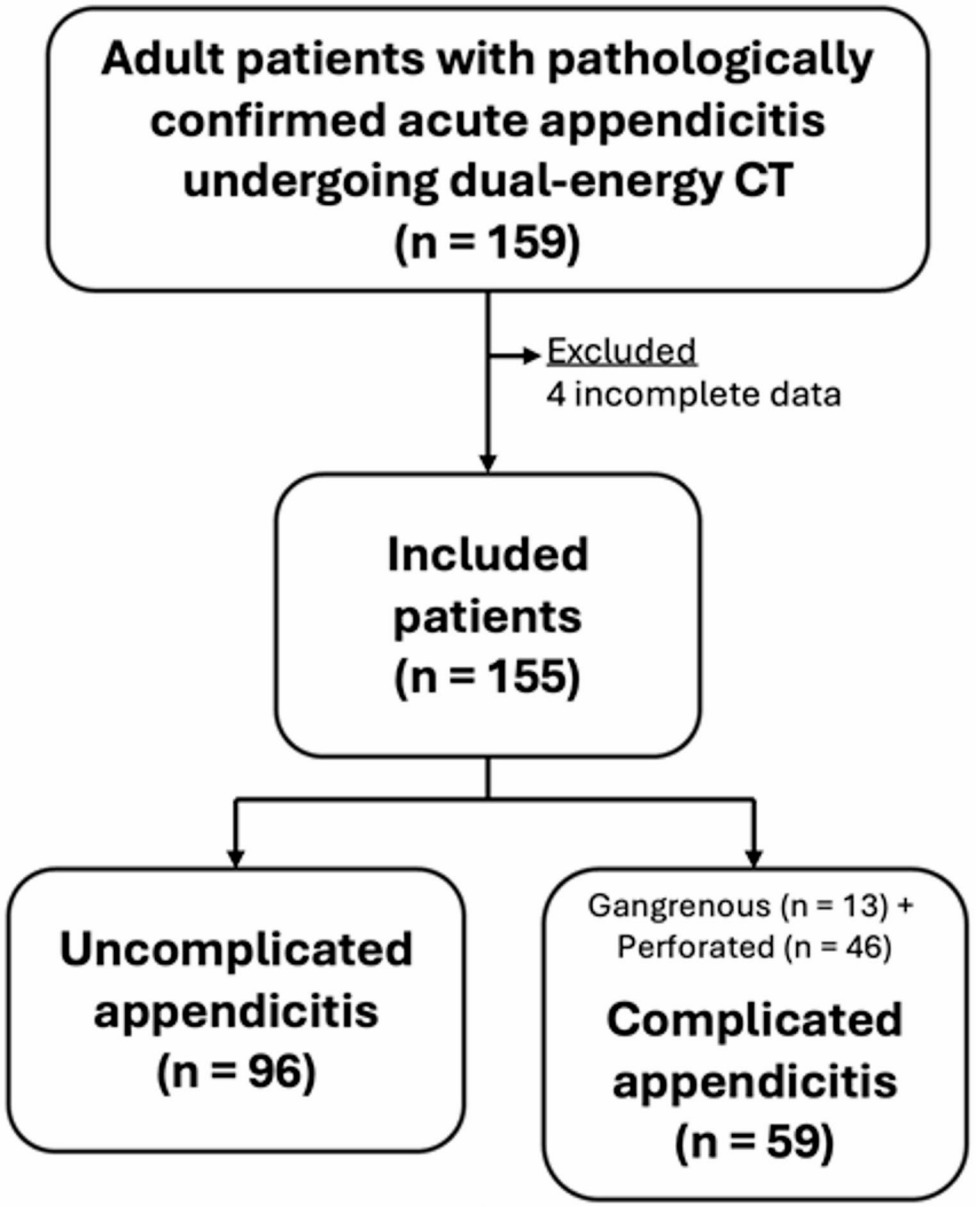
Flowchart of patient inclusion

This single-center, retrospective, descriptive investigation was conducted at a 2200-bed urban academic medical center. Institutional Review Board approval was obtained (certificate of approval number Si 161/2024) with a waiver of informed consent due to the study’s retrospective nature. Eligible patients were identified through a hospital database search for International Classification of Diseases, 10th revision (ICD-10) codes related to acute appendicitis and appendectomy, including all consecutive inpatients aged 18 years or older from February 2020 to February 2024 who underwent surgery within 24 h of DECT (*n* = 159). Those with incomplete data (*n* = 4) were excluded. The final study cohort comprised 155 patients, all of whom had pathologically confirmed diagnosis of acute appendicitis. Sample size was based on all available patients meeting inclusion criteria during the study window. No formal power calculation was performed. All diagnoses and procedures were verified by chart review to confirm coding accuracy. Note that the study included 17 patients from a previously reported cohort performed for the evaluation of appendicoliths [12] (Fig. 1).

### DECT acquisition

All DECT examinations were performed using one of the three available single-source, rapid-kVp-switching DECT scanners (Revolution CT, Revolution Apex, GE Healthcare). The protocol included at least a portovenous phase, acquired 70–80 s after intravenous contrast administration. Scan parameters were as follows: tube potential of 80–140 kVp, tube current of 280 mA, helical pitch of 0.992:1, scan rotation time of 0.6 s, and slice thickness of 1.25 mm, covering the region from diaphragmatic domes or upper poles of both kidneys to the ischial tuberosities. Contrast media were administered via an injector at an infusion rate of 2–3 mL/s, with a total volume of 1.5-2.0 mL/kg. Images were postprocessed using a dedicated workstation (AW 4.7, GE Healthcare) and included virtual monochromatic (VMI) images at 50 keV (“low keV”) and 70 keV (surrogates for the 120-kVp-equivalent portovenous phase). These images were reconstructed at slice thickness of 1.25-mm. All images were archived in the Picture Archiving and Communication System (PACS) for interpretation. To evaluate radiation exposure, a random subset of 40 studies was reviewed. The median volume CT dose index (CTDIvol) was 7.15 mGy (range: 6.56–13.86 mGy), and the median dose-length product (DLP) was 354.60 mGy-cm (range; 227.52–737.00 mGy-cm).

### Image re-interpretation and definitions of CT findings

**Fig. 2:**
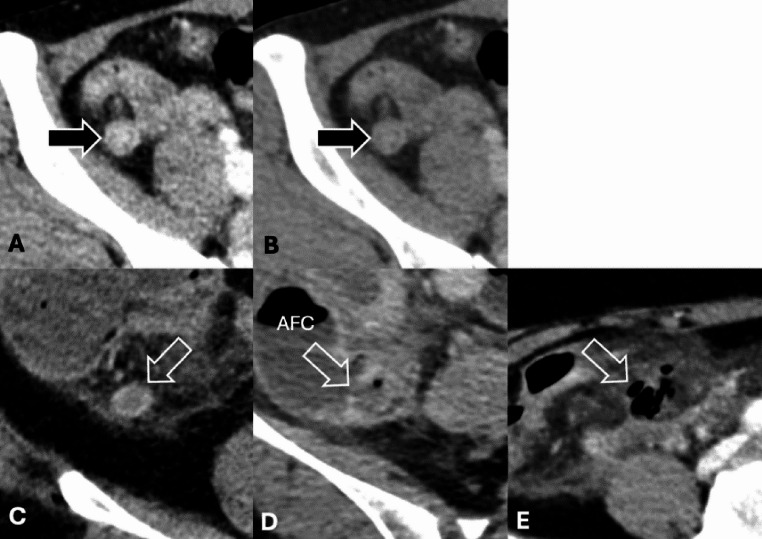
Wall enhancement patterns of acute appendicitis. Axial contrast-enhanced CT images (A: 50-keV virtual monoenergetic, B: 120-kVp-equivalent) show smooth, uninterrupted enhancement of the wall (black arrows). Note an apparent increase in iodine attenuation on the low-keV image, relative to conventional image. Figure 2C, D and E are 50-keV images of three patients depicting interrupted appendiceal wall enhancement (i.e., defect; open arrows) due to nonenhancing wall, fluid in the wall, and air in the wall, respectively. *AFC* air-fluid collection

**Fig. 3:**
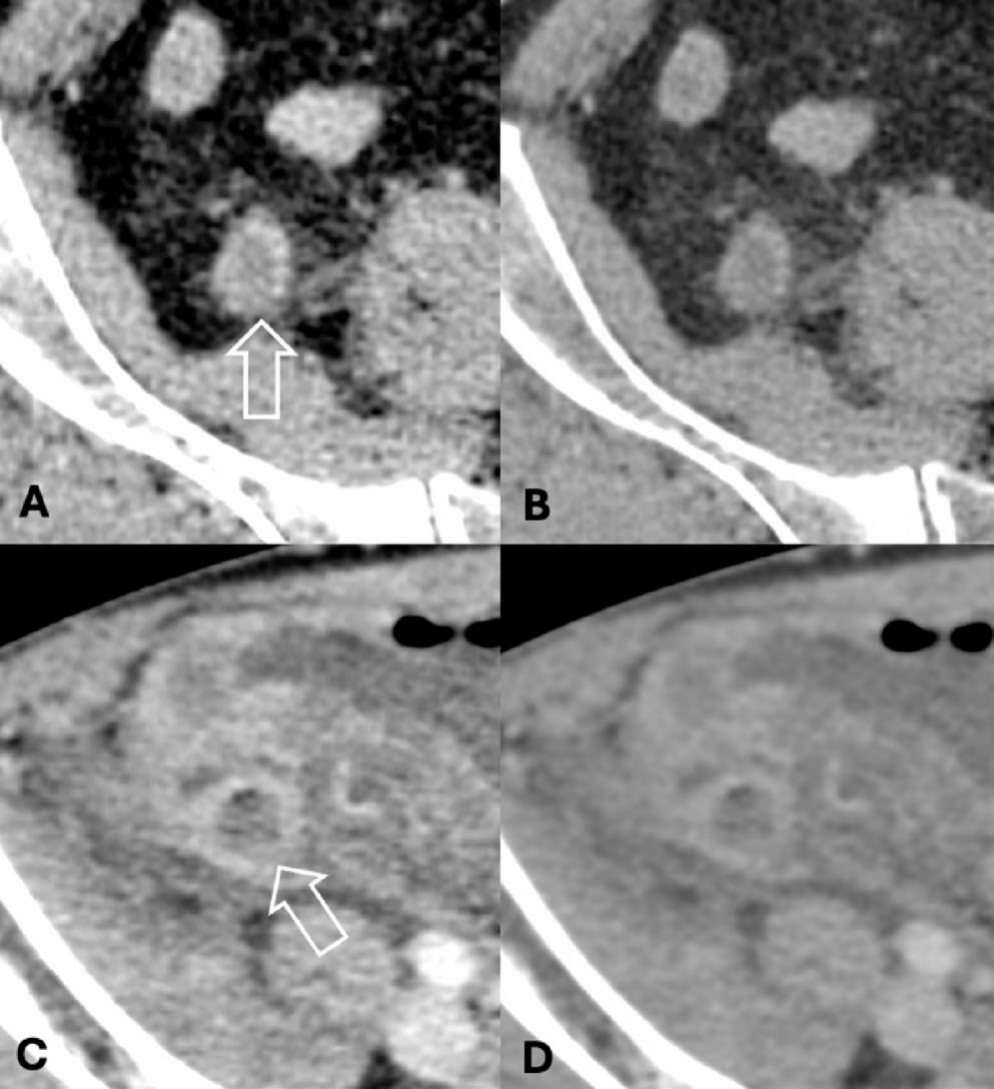
Value of low-keV virtual monoenergetic images (VMI) in depicting wall enhancement defects of acute appendicitis. Examples of two cases where wall enhancement defects (open arrows) were better appreciated on 50-keV VMI (A and C), while not apparent on 120-kVp-equivalent images (B and D)

Two radiologists independently re-reviewed all DECT scans (one subspecialized in abdominal imaging with 14 years of experience and another in emergency imaging with 9 years of experience), blinded to clinical and pathological data except for the diagnosis of appendicitis and provided definitions of CT findings (**Supplementary E1**). The re-interpretation was conducted in two separate rounds. In the first round, only 50-keV VMI images were assessed, with particular focus on identifying absent or decreased enhancement of the inflamed appendiceal wall (“wall enhancement defect”; Figs. 2 and 3). The second round, conducted 4 weeks later, involved a review of the 120-kVp-equivalent portovenous images for findings related to acute appendicitis, including the wall enhancement defect. In the final step of the second round, radiologists re-review the 50-keV VMI images alongside the 120-kVp-equivalent portovenous images to assess for wall enhancement defect.

In each round, radiologists classified appendicitis severity as uncomplicated and complicated. They also provided confidence levels for the presence of wall enhancement defect, using a four-tier scale: 25–49% (low confidence), 50–74% (moderate confidence), 75–90% (high confidence), and > 90% (very high confidence). All discrepancies were resolved by a third radiologist (RK), an emergency radiologist with 24 years of experience, utilizing standard definitions (Supplementary Table E1) while the discrepancies of confidence levels were re-scored. No indeterminate or missing image interpretations occurred.

### Reference standards

The diagnosis of acute appendicitis was confirmed by histopathological analysis. Complicated appendicitis encompassed those with gangrene or perforation. Uncomplicated and gangrenous appendicitis was identified through histopathological evaluation, while perforation was diagnosed based on operative findings or histopathological reports.

### Statistical analysis

Categorical variables, including sex, symptoms, signs, and CT findings, were summarized as frequencies and percentages. Continuous variables, such as age, body mass index, temperature, time from visit to CT, duration of symptoms, and time to antibiotics, were presented as mean ± standard deviation for normally distributed data or median (range) for non-normally distributed data.

The diagnostic performance of DECT in classifying the severity of acute appendicitis was assessed using surgical and histopathological findings as the reference standard. To compare the detection rates of appendiceal wall enhancement defects across the three imaging series, 50 keV, 120-kVp-equivalent, and the combination of 50 keV and 120-kVp-equivalent, Cochrane’s Q test was employed, which is suitable for assessing differences in proportions across related samples. If a significant result was obtained, pairwise comparisons were conducted using McNemar’s test, with adjustments for multiple comparisons (e.g., Bonferroni correction). These analyses were performed separately for all patients, as well as for subgroups with uncomplicated and complicated appendicitis.

Comparative analyses were performed between patients with uncomplicated and complicated appendicitis. Categorical variables were compared using either Yates’ continuity-corrected chi-square test or Fisher’s exact test, as appropriate. For continuous variables, the independent samples t-test was used for normally distributed data, and the Mann-Whitney U test for non-normally distributed data. Univariable logistic regression analyses were performed to identify potential predictors. Variables with p-values < 0.1 in the univariable analysis were subsequently entered into a multivariable logistic regression model using the forward stepwise selection method based on the likelihood ratio test (SPSS, PIN = 0.05, POUT = 0.10). Associations were quantified using odds ratios with corresponding 95% confidence intervals.

All statistical analyses were conducted using IBM SPSS Statistical Package for the Social Science for Windows, version 27, the R DescTools package, and CIA software, version 2.2.0. A two-tailed p-value of less than 0.05 was considered statistically significant.

## Results

The patient cohort (Table 1) consisted mostly of females with a mean age of 47.4±19.0 years, a median symptom duration of 24 h (range: 1–240), a median Alvarado score of 6 (range: 0–10) and a median time to CT of 5.5 h (range: 1.2–43.1).

**Table 1:** Comparison of patients’ demographics, clinical characteristics and computed tomographic (CT) findings between uncomplicated and complicated appendicitis (*n* = 155)

Factors	All patients (*n* = 155)	Uncomplicated appendicitis (*n* = 96)	Complicated appendicitis (*n* = 59)	*P* values
Mean age (SD)	47.4 (19.0)	41.1 (17.6)	57.7 (16.6)	*< 0.001*
Male sex (%)	50 (32.3)	29 (30.2)	21 (35.6)	0.603
Mean body mass index (SD) (n = 152)	23.6 (4.0)	23.4 (4.3)	23.8 (3.6)	0.651
Median durations (hr; range)				
From symptoms to arrival (*n* = 151)	24.0 (1.0-240.0)	24.0 (1.0-240.0)	24.0 (3.0-168.0)	*0.003*
From symptoms to CT (*n* = 151)	5.5 (1.2–43.1)	5.5 (1.2–27.5)	6.1 (3.2–43.1)	0.180
From arrival to antibiotics (*n* = 138)	7.1 (0.4–28.4)	7.2 (2.4–28.4)	6.8 (0.4–16.2)	0.371
Symptoms and signs				
Right lower quadrant pain (%) (*n* = 152)	146 (96.1)	90 (95.7)	56 (96.6)	1.000
Mean body temperature (^0^C; %) (*n* = 151)	36.8 (2.7)	36.5 (3.3)	37.2 (0.71)	0.177
Rebound tenderness (%) (*n* = 152)	72 (47.4)	43 (45.7)	29 (50.0)	0.731
Migratory pain (%) (*n* = 152)	65 (42.8)	42 (49.4)	23 (34.3)	0.089
Anorexia (%) (*n* = 152)	54 (35.5)	29 (30.9)	25 (43.1)	0.174
Nausea and vomiting (%) (*n* = 152)	69 (45.4)	38 (40.4)	31 (53.4)	0.162
Laboratory results				
Median white blood cell counts (mm^3^; range) (*n* = 154)	12,635 (120 − 28,490)	12,670 (1,060 − 28,490)	12,570 (120 − 26,720)	0.339
% median neutrophil (range) (*n* = 154)	83.0 (2.0–94.0)	82.5 (2.0–93.0)	83.8 (8.4–94.0)	0.499
Median absolute neutrophils (*n* = 154)	10,540.3 (10.1-24769.4)	10542.520 (21.2-20.468)	10,432.8 (10.1, 24,769.4)	0.443
Median Alvarado score (*n* = 152)	6 (0, 10)	6 (1–10)	6 (0, 10)	0.383
CT findings				
Median maximum diameter (mm; range)	11.5 (4.9, 23.9)	10.1 (5.9–22.3)	13.2 (4.9–23.9)	*< 0.001*
Median tip diameter (mm; range)	9.7 (4.2–21.0)	8.7 (4.2–21.0)	11.5 (4.9–21.0)	*< 0.001*
Appendicolith (%)	55 (35.5)	18 (18.8)	37 (62.7)	*< 0.001*
Periappendiceal fat stranding (%)	140 (90.3)	81 (84.4)	59 (100)	*0.004*
Periappendiceal fluid (%)	71 (45.8)	33 (34.4)	38 (64.4)	*0.001*
Periappendiceal fluid collection (%)	26 (16.8)	2 (2.1)	24 (40.7)	*< 0.001*
Ascites (%)	35 (22.6)	17 (17.7)	18 (30.5)	0.098
Extraluminal air (%)	19 (12.3)	0 (0)	19 (32.2)	*< 0.001*
Small bowel wall thickening (%)	43 (27.7)	10 (10.4)	33 (55.9)	*< 0.001*
Small bowel dilatation (%)	35 (22.6)	13 (13.5)	22 (37.3)	*0.001*
Appendiceal wall enhancement defect on… (%)				
50 keV	77 (49.7)	26 (27.1)	51 (86.4)	*< 0.001*
120-kVp equivalent	58 (37.4)	10 (10.4)	48 (81.4)	*< 0.001*
Combined 50 keV and 120-kVp equivalent	64 (41.3)	15 (15.6)	49 (83.1)	*< 0.001*

*SD* standard deviation

Significant values are in *italics*

Of these patients, 59 had complicated disease (46 perforated and 13 gangrenous nonperforated cases). The diagnostic performance of the three viewing methods for differentiating complicated from uncomplicated appendicitis-based on radiologists’ overall impressions-showed sensitivities5 pe ranging from 81.4 to 89.8% (highest with 50-keV images alone) and specificities from 76.0 to 87.5% (highest with 120-kVp-equivalent images alone). Combining 50-keV and 120-kVp-equivalent images yielded balanced sensitivity and specificity of 83.1% and 86.5%, respectively (Table 2).

**Table 2:** Diagnostic performance of radiologists’ impression in the differentiation between uncomplicated and complicated appendicitis with surgical and pathological final diagnoses as a reference standard (*n* = 155)

	Radiologists’ impression utilizing all CT findings on… image series		
	50 keV	120-kVp equivalent	Combined 50 keV and 120-kVp equivalent
True positive	53	48	49
False positive	23	12	13
True negative	73	84	83
False negative	6	11	10
Sensitivity (%; 95% CI)	89.8 (79.5–95.3)	81.4 (69.6–89.3)	83.1 (71.5–90.5)
Specificity (%; 95% CI)	76.0 (66.6–83.5)	87.5 (79.4–92.7)	86.5 (78.4–91.9)
Positive predictive value (%; 95% CI)	69.7 (58.7–78.9)	80.0 (68.2–88.2)	79.0 (67.4–87.3)
Negative predictive value (%; 95% CI)	92.4 (84.4–96.5)	88.4 (80.4–93.4)	89.2 (81.3–94.1)
Accuracy (%; 95% CI)	81.3 (74.4–86.6)	85.2 (78.7–89.9)	85.2 (78.7–89.9)

*CI* confidence interval

Wall enhancement defects were most frequently identified using 50-keV images alone (48/122; 39.3%), followed by combined 50-keV and 120-kVp-equivalent images (35/122; 28.7%), and 120-kVp-equivalent images alone (29/122; 23.8%). Radiologists’ confidence in detecting this finding was highest when using the combined 50-keV and 120-kVp-equivalent images in all groups (Table 3).

**Table 3:** Subgroup analysis of 122 patients without extraluminal air or periappendiceal fluid collection on DECT

Number of	50 keV	120-kVp equivalent	Combined 50 keV and 120-kVp equivalent	*P* values
Appendiceal wall enhancement defect (%)				
All patients (*n* = 122)	**48 (39.3)**	29 (23.8)	35 (28.7)	*< 0.001*
Uncomplicated appendicitis (*n* = 94)	**24 (25.5)**	9 (9.6)	14 (14.9)	*< 0.001*
Complicated appendicitis (*n* = 28)	24 (85.7)	20 (71.4)	21 (75.0)	0.039^a^
Confidence level > 90% (%)				
All patients (*n* = 122)	88 (72.1)	89 (73.0)	**112 (91.8**)	*< 0.001*
Uncomplicated appendicitis (*n* = 94)	*66 (70.2)*	*75 (79.8)*	*88 (93.6)*	*< 0.001*
Complicated appendicitis (*n* = 28)	22 (78.6)	**14 (50.0)**	24 (85.7)	*< 0.001*

Comparison of detection rates of wall enhancement defects and radiologists’ confidence levels using 50 keV, 120-kVp-equivalent, and combined 50 keV and 120-kVp-equivalent image series in all patients, those with uncomplicated, and complicated appendicitis

*keV* kiloelectron volt, *kVp* kilovoltage peak

The values in bold indicate values that exhibit a significant difference from the other two images in a pairwise comparison

The values in italics indicate same-row pairs that exhibit a significant difference in a pairwise comparison

^a^All pairwise comparisons fail to reach significance (*p* > 0.05)

Four factors were independently associated with complicated appendicitis (Table 4): age > 40 years, wall enhancement defects, fluid collection, and extraluminal air.

**Table 4:** Univariable and multivariable logistic regression analyses of predictive factors of complication in adults who had acute appendicitis

	Univariable model		Multivariable model	
Factors	Unadjusted OR (95% CI)	*P* Value	Adjusted OR (95% CI)	*P* Value
Age > 40	7.534 (3.231–17.567)	*< 0.001*	5.399 (1.672–17.436)	*0.005*
Wall enhancement defect on combined 50 keV and 120-kVp equivalent	26.460 (11.027–63.490)	*< 0.001*	10.820 (3.649–32.081)	*< 0.001*
Periappendiceal fluid collection	32.229 (7.236-143.544)	*< 0.001*	11.957 (0.893–8.245)	*0.015*
Extraluminal air	NA		NA	

*CI* confidence interval, *OR* odds ratio

NA = not applicable (because of no extraluminal air in the uncomplicated appendicitis group)

Significant values are in *italics*

A subgroup analysis of appendicitis without fluid collection or extraluminal air revealed that age > 40 years and wall enhancement defects on combined 50-keV and 120-kVp-equivalent images were independent predictors of complicated appendicitis, with odds ratios of 6.012 and 9.312, respectively (Table 5).

**Table 5:** Subgroup analysis of 122 patients without extraluminal air or periappendiceal fluid collection on DECT

	Univariable model		Multivariable model	
Factors	Unadjusted OR (95% CI)	*P* Value	Adjusted OR (95% CI)	*P* Value
Age > 40	7.429 (2.390-23.087)	*< 0.001*	6.012 (1.520-23.785)	*0.011*
Wall enhancement defect on combined 50 keV and 120-kVp equivalent	17.143 (6.140–47.860)	*< 0.001*	9.312 (2.470-35.101)	*< 0.001*

Univariable and multivariable logistic regression analyses of predictive factors of complication in adults who had acute appendicitis

*CI* confidence interval, *OR* odds ratio

Significant values are in *italics*

## Discussion

We identified that among the three methods of image viewing, combining 50-keV and 120-kVp-equivalent images achieved the most balanced diagnostic performance-83.1% sensitivity and 86.5% specificity-for distinguishing complicated from uncomplicated appendicitis, with sensitivity slightly lower than 50-keV images alone. Additionally, wall enhancement defects remained independently predictive of complicated appendicitis even among cases without fluid collection or extraluminal air, highlighting their diagnostic importance in subtle or early presentations.

### Diagnostic performance of 50-keV images, 120-kVp-equivalent images, and a combination in the differentiation of uncomplicated and complicated appendicitis

Single-energy CT has shown variable diagnostic performance in this differentiation. A meta-analysis by Bom WJ et al. [8] reported a pooled sensitivity of 78% and specificity of 91%, while a prospective study reported 55% sensitivity, dropping to 36% in the low-dose (< 2 mSv) CT protocol [7]. Conditional CT pathways (e.g., ultrasound first, followed by CT if negative)showed sensitivities of 38–45% and specificities of 68–81% [9], reflecting suboptimal performance in real-world settings.

In contrast, recent review articles highlighted the value of low keV VMI DECT in gastrointestinal imaging [13, 14]. Lev-Cohain N et al. [11] demonstrated improved detection of inflamed appendices with 50-keV images using both quantitative (higher attenuation values, signal-to-noise (SNR) and contrast-to-noise (CNS) ratios) and qualitative metrics. Elbanna KY et al. [10] reported excellent performance (100% sensitivity, 81.2% specificity, and 85.2% accuracy) in differentiating gangrenous from simple appendicitis, using 40 keV VMI, with outstanding interobserver agreement (k = 0.99). Our study similarly found high diagnostic values, with sensitivities of 81.4–89.8% and specificities of 76.0-87.5%, consistent with these findings.

### Significance of wall enhancement defects in the subset of appendicitis without Obvious signs of perforation

Wall enhancement defects, indicating gangrenous change, are a key imaging sign of complicated appendicitis. A meta-analysis by Kim HY et al. [15] found pooled sensitivity of 59% and specificity of 96%. It is one of the few CT signs independently predictive of complicated disease [16, 17, 18, 19, 20, 21, 22, 23, 24]. Our investigation confirms its value in both the overall cohort and in the specific subgroup lacking overt signs of perforation (i.e., fluid collection, extraluminal air), where it emerged as the most reliable indicator of complication.

In our study, this sign was most frequently detected using low-keV images. However, radiologist confidence was highest with the combined 50-keV and 120-kVp-equivalent series. This likely reflects reader familiarity with the conventional 120-kVp equivalent appearance, highlighting the potential benefit of dual-viewing to leverage both familiarity and enhance conspicuity.

### Factors predictive of complicated appendicitis

Our study identified four independent predictors of complicated appendicitis: age > 40 years, wall enhancement defects, fluid collection, and extraluminal air. These findings are consistent with previously published literature, suggesting stability in the disease’s imaging characteristics despite evolving technology.

A wide range of clinical and imaging predictors has been reported in the past decade. Most consistent across studies are CT features such as extraluminal air, fluid collection, as well as wall enhancement defects. Some features, like appendicolith, appendix diameter, and fat stranding, show variable significance depending on study design, population, or statistical approach. Summary of findings and their reported predictor value in the literature:

- Older age– consistently significant [16, 25, 26, 27, 28, 29, 30, 31].
- Fluid collection and extraluminal air - strong indicators of with complicated appendicitis [16, 17, 18, 25, 26, 30, 33, 34], suggesting perforation [10, 35, 36].
- Wall enhancement defects– consistently significant [16, 17, 18, 33].
- Appendix diameter [16, 17, 18, 22, 26, 31, 32, 34, 37, 38, 39], appendicolith [12, 16, 19, 25, 28, 31, 32, 33, 38, 40, 41, 42], periappendiceal fat stranding [16, 18, 21, 23, 31, 37, 40] and periappendiceal fluid [26, 28, 30, 32]– showing mixed results depending on analysis level.

Differences in may be due to factors such as sample size, statistical power, variatble inclusion in multivariable models, and differing imaging techniques or interpretation thresholds.

The study has several limitations. It was a retrospective, single-institution study with a relatively small sample size, limiting the generalizability of findings. Only patients who underwent surgery with pathologically confirmed appendicitis and dual-energy CT were included, restricting applicability to this subset. A large proportion of patients in our cohort underwent single-energy CT due to lack of standardized protocols. However, findings in this study were comparable to previous research in identifying features associated with complicated appendicitis. All three interpreting radiologists were expert, which may have contributed to the high diagnostic performance observed and limited the generalizability of the findings. Trainees were not involved in the interpretation, leaving room for future studies to assess whether low-keV imaging maintains its value among less experienced readers. There is a lack of formal inter-observer agreement statistics. However, all imaging features were initially reviewed independently by two experienced subspecialty radiologists, with discrepancies resolved by a third expert adjudicating discordant cases. While this structured consensus approach was employed to ensure consistent and clinically relevant interpretations, particularly for subjective findings such as wall enhancement defects, it may limit the generalizability of our findings to less experienced readers. However, in theory, the enhanced contrast provided by low-keV imaging– particularly the improved conspicuity of vascular and wall enhancement– may offer even greater benefit for less experienced radiologists by making subtle findings more visually apparent. Further studies involving readers with varying levels of experience are warranted to explore this possibility. We also did not evaluate the use of color iodine-overlay maps due to workflow limitations. Prior research has shown these maps provide similar benefits to low keV imaging [10].Wall enhancement defects remain a subjective finding, likely influenced by individual radiologists’ thresholds. This may explain why some patients with pathologically confirmed uncomplicated appendicitis were still noted to have this sign. Nevertheless, it appears to be a useful feature, especially in cases lacking other CT indicators of perforation. Finally, one important consideration in the use of dual-energy CT is radiation exposure. While DECT was traditionally thought to incur higher doses, advances in technology have enable dose-neutral or even dose-reducing protocols compared to conventional single-energy CT and at the same time offering added diagnostic benefits including material decomposition and improved tissue contrast. In our study, radiation dose was not the primary focus but the protocol used was optimized for abdominal imaging within accepted dose limits.

In conclusion, low-keV images should be interpreted alongside 120-kVp equivalent images to optimize detection of wall enhancement defects in patients with CT-diagnosed acute appendicitis, particularly for identifying complications in the absence of clear perforation.

## Electronic supplementary material

Below is the link to the electronic supplementary material.

Supplementary Material 1

## Data Availability

No datasets were generated or analysed during the current study.

## Abbreviations

DECT: dual-energy CT
keV: kiloelectron volt
kVp: kilovoltage peak
PACS: Picture Archiving and Communication Systems
VMI: virtual monoenergetic image
