# Supplementary material for: Low-keV virtual monoenergetic images with rapid kilovoltage-switching DECT for differentiating complicated from uncomplicated appendicitis in adults
Source: Abdom Radiol (NY). 2025 Jul 17;51(2):630–8. doi: 10.1007/s00261-025-05124-2 (PMC12929296; doi:10.1007/s00261-025-05124-2)
Supplement: Supplementary file 1 — Supplementary Material 1 [file 261_2025_5124_MOESM1_ESM.docx]

**Supplementary Table E1**: Definitions of CT findings

|  | **Definitions** |
| --- | --- |
| Appendix diameter | Outer-to-outer wall shortest diameter of appendix measured in the axial CT images that were perpendicular to the appendix’s axis. |
| Wall enhancement defect | Interruption of wall enhancement of appendicitis due to decreased or absent enhancement with or without intervening fluid or air attenuation |
| Appendicolith | A hyperattenuating focus with a diameter >2 mm located either inside the appendiceal lumen or outside in fluid or fluid collection, shown on an unenhanced phase |
| Periappendiceal fat stranding | Increased attenuation of fat surrounding appendix of >2 mm in thickness |
| Periappendiceal fluid | Extraluminal fluid around the appendix without encapsulation |
| Periappendiceal fluid collection | Extraluminal fluid around the appendix with encapsulation |
| Ascites | Free fluid, which is considered larger than a physiologic amount |
| Small bowel wall thickening | Thickness of small bowel loops (near the appendix) of greater than 3 mm providing that the loop is distended |
| Small bowel dilatation | Transverse diameter of small bowel loops (near the appendix) of greater than 2.5 cm |
| Extraluminal air | Extraluminal air around the appendix or elsewhere in the abdomen and pelvis presumed to be related to appendicitis |
